# Occupational cancer in developed countries

**DOI:** 10.1186/1476-069X-10-S1-S9

**Published:** 2011-04-05

**Authors:** Aaron Blair, Loraine Marrett, Laura Beane Freeman

**Affiliations:** 1 Occupational and Environmental Epidemiology Branch, Division of Cancer Epidemiology and Genetics, National Cancer Institute, Bethesda, MD, USA; 2 Occupational Cancer Research Centre, Cancer Care Ontario, Toronto, ON, Canada

## Abstract

**Abstract:**

Studies of occupational exposures have made major contributions to our understanding of human carcinogenesis. About one third of the factors identified as definite or probable human carcinogens were first investigated in the workplace and these exposures exact a considerable toll on working populations. There are many additional workplace exposures that are suspect carcinogens that require further evaluation to ensure a safe work environment. Information from occupational investigations is also relevant to the general population because many occupational exposures can be found outside the workplace. Much of our understanding about occupational cancer has been obtained from studies largely composed of white men in developed countries. The movement of industry from developed to developing countries underscores the need for future investigations to include more diverse populations.

## What do we know?

Studies of exposures in the workplace have made major contributions to our understanding of human carcinogenesis. The International Agency for Research on Cancer (IARC) Monographs on the Evaluation of Carcinogenic Risks to Humans, which were first published in 1972 [1]when Lorenzo Tomatis was the Chief of the Unit of Chemical Carcinogenesis, provide reviews of occupational exposures and other factors for possible carcinogenicity. From IARC Monograph evaluations through 2003, occupational factors represent 31%, 42%, and 42%, respectively, of factors classified as Sufficient, Probable, and Possible human carcinogens [2]. Since 2003, occupational factors continue to be highly prevalent (about 50%) in new or upgraded IARC classifications. For example, shift work, which was evaluated for the first time in 2007 is listed as a probable human carcinogen and titanium dioxide and talc as possible human carcinogens. In addition, occupational exposures that have been upgraded include formaldehyde and butadiene (to sufficient), various cobalt and lead compounds (to probable), and carbon black (to possible) (see International Agency for Research on Cancer website for these results (http://monographs.iarc.fr/)). Most of the early epidemiologic studies on occupational cancer came from studies in developed countries, but more recently studies in developing countries are becoming more common place. Given the prominent role played by occupational exposures in our understanding of human carcinogenesis, it is worthwhile to take stock of what we know about occupational cancer, what we are doing now, how successful this enterprise has been in reducing the burden of cancer, and what we should be doing in the future.

Classic human carcinogens first identified in the occupational environment such as arsenic, asbestos, benzene, benzidine, chromium, mustard gas, nickel, radon, and vinyl chloride, document the contribution of occupational studies to our understanding of human carcinogenesis [3]. In addition to these well-established carcinogens, there is a very long list of workplace exposures that are suspected human carcinogens that need further evaluation [4]. Occupational carcinogens also have an impact beyond the workplace because many, probably most, are also found in some non-occupational settings. Occupational carcinogens affect many cancer sites. Airway sites are most common, but bladder and skin are frequent [3]. There are a number of suggested associations between lymphatic and hematopoietic cancers and occupational exposures, but links with the cancers of the digestive system are relatively infrequent [2]. Many occupations or occupational carcinogens show definite and probable links with more than one cancer site. For example, asbestos is associated with mesothelioma and cancers of the lung, larynx, and gastrointestinal tract; chromium with cancers of the nasal cavities and lung; arsenic with cancers of the liver, lung and skin, mustard gas with cancers of the pharynx, larynx, and lung; employment in coking plants with cancers of the lung, skin, and bladder; ionizing radiation with leukemia and cancers of the liver, lung, bone, breast, and thyroid; and soots with cancers of the esophagus, lung, and skin [3]. In summary, studies of workplace exposures have made major contributions to our understanding of the etiology of cancer.

## Occupational methods - what we are doing now and what should we do in the future?

Much of the occupational cancer research in the past has focused on white men in developed countries [5]. To get some indication of the nature of occupational cancer research today, we surveyed papers published in the American Journal of Industrial Medicine from January, 2007 through June, 2009. Of the 256 articles published during this time period, 31 (12%) were on cancer. Of the papers on cancer, 27 were from developed countries and 24 included white men, 12 included women, and 3 included minorities (a few papers included more than one of these groups). Although this might be a biased sample because it is located in the United States, these data suggest that the pattern of a predominance of studies of white men noted by Zahm et al. [5] may still persist and that enhanced efforts are needed to incorporate other populations into occupational cancer studies. With the movement of many industries from the developed to developing countries it becomes even more important to significantly expand research on occupational cancer in these areas.

There is a need to incorporate quantitative exposure assessment in occupational studies to more clearly assess disease-exposure relationships and to provide critical information for regulatory decision-making [6-8]. Our brief survey found that only one paper included quantitative exposure assessments, while 18 papers exposures were based simply on occupation/industry titles. Apparently quantitative assessments are still relatively rare in occupational studies of cancer. Reliance upon cruder exposure assessments results in greater misclassification, which would diminish observed relative risks and lead to false negative conclusions [9]. The impact of crude exposure classification is evident from data on established carcinogens. For example, the relative risk of lung cancer in the classic British doctors study ranges from 5.6 for regular smokers of 1 to 4 cigarettes per day to 50.7 for the smokers of 35 to 40 cigarettes [10]. A simple ever/never categorization of persons as either smokers or non-smokers, however, produces a relative risk of 12.0, a technique often found in occupational studies, which greatly underestimates the risk at higher levels of exposure [10]. Although the lung cancer risk from smoking is not hidden when using a simple ever/never classification of exposure, it might be for other exposures where the relative risk in the upper exposure category is five rather than 50. In addition, studies of established occupational carcinogens typically show that risk estimates are smaller from use of more simplistic exposure metrics, e.g., duration of exposure than from estimates of intensity [6].

Future epidemiologic efforts should include quantitative approaches to exposure assessment to diminish the chances of false negative findings from excessive exposure misclassification and to accurately characterize the exposure-response relationship. Quantitative exposure assessment is a challenge. Availability of quantitative assessments of exposure also provide the opportunity for more constructive evaluation of gene-exposure and lifestyle-exposure interactions, which help to more fully characterize subgroup risk and mechanistic pathways.

## Prevention - are occupational cancers controlled through intervention?

Most countries have undertaken efforts to prevent occupational cancer through control of carcinogenic exposures. Although these efforts have undoubtedly reduced the number of cancers from some exposures, the overall impact of this intervention has not been well documented. Tomatis et al. [11] addressed this question over a decade ago and concluded that this is not a research area that had received much attention, but it should. Demonstration of a reduction in cancer from control of workplace exposures is complicated by cancer latency which might require a considerable time lapse after exposure intervention before a reduction in cancer is observed. For example, reduction in lung cancer after smoking cessation is not visible until five years after cessation and a 50% reduction is not achieved for 15 years [12].

There are success stories. Scrotal cancer in England and Wales showed a remarkable decline from personal hygiene intervention [13]. Nasal cancer is considerably less frequent among furniture workers first employed after 1940 when exposure to wood dust was reduced than among those employed earlier [14]. Sweden passed regulations to restrict exposure to asbestos in the mid-1970s, making it one of the first countries to do so. By the 1990s, the incidence of pleural mesothelioma had stabilized [15].

## Conclusions

Although the number of known and suspected occupational carcinogens is large and continues to grow, it appears that the current scientific effort is not keeping pace with the need. Despite a growing list of suspected carcinogens with insufficient information for conclusive evaluation, the effort to identify occupational carcinogens (i.e., the number in the 2A and 2B IARC categories) has tapered off. From the WHO rating of carcinogens in 1964 to the IARC Supplement 7 evaluation in 1982, the number of suspected occupational carcinogens increased dramatically from 9 to 92 (about 1000%) [2] while between the IARC evaluations of 1982 and 2003, the number increased only from 92 to 137 (about 50%). Not only is this a much smaller percentage increase, but also a much smaller absolute number increase (i.e., 45 versus 83). This could reflect a decrease in the number of occupational exposures remaining to be identified as carcinogens. However, given the very large number of chemicals in the workplace, the changing work environment, and the constant addition of new exposures, this seems unlikely. More likely this diminishing number of newly identified carcinogens reflects a diminished scientific effort. It is our the impression that the number of investigators engaged in the study of occupational cancer has decreased considerably over the past two or three decades. The number of papers on occupational cancer in general epidemiology meetings seems considerable fewer than 30 years ago. Research units in academia, government, and industry focusing on occupational cancer may have also diminished. In public health research, there are cycles of focus and popularity. Typically, however, previous success, the opportunity for advancement of scientific understanding, and public health need are the critical determinants of popularity. The success of occupational studies in identifying hazards and in elucidating carcinogenic processes, the large number workplace exposures requiring further evaluation, the high levels of exposure which are very useful for mechanistic studies, and the exposure of the general public to many of these same chemicals, would seem to make a strong argument for a significant public health effort on cancer in the workplace, but this appears not to have been the case in recent years.

## Competing interests

The authors declare that they have no competing financial or non-financial interests.
